# Advancements in Imaging Sensors and AI for Plant Stress Detection: A Systematic Literature Review

**DOI:** 10.34133/plantphenomics.0153

**Published:** 2024-03-01

**Authors:** Jason John Walsh, Eleni Mangina, Sonia Negrão

**Affiliations:** ^1^ School of Biology & Environmental Science, University College Dublin, Belfield, Dublin, Ireland.; ^2^ School of Computer Science, University College Dublin, Belfield, Dublin, Ireland.

## Abstract

Integrating imaging sensors and artificial intelligence (AI) have contributed to detecting plant stress symptoms, yet data analysis remains a key challenge. Data challenges include standardized data collection, analysis protocols, selection of imaging sensors and AI algorithms, and finally, data sharing. Here, we present a systematic literature review (SLR) scrutinizing plant imaging and AI for identifying stress responses. We performed a scoping review using specific keywords, namely abiotic and biotic stress, machine learning, plant imaging and deep learning. Next, we used programmable bots to retrieve relevant papers published since 2006. In total, 2,704 papers from 4 databases (Springer, ScienceDirect, PubMed, and Web of Science) were found, accomplished by using a second layer of keywords (e.g., hyperspectral imaging and supervised learning). To bypass the limitations of search engines, we selected OneSearch to unify keywords. We carefully reviewed 262 studies, summarizing key trends in AI algorithms and imaging sensors. We demonstrated that the increased availability of open-source imaging repositories such as PlantVillage or Kaggle has strongly contributed to a widespread shift to deep learning, requiring large datasets to train in stress symptom interpretation. Our review presents current trends in AI-applied algorithms to develop effective methods for plant stress detection using image-based phenotyping. For example, regression algorithms have seen substantial use since 2021. Ultimately, we offer an overview of the course ahead for AI and imaging technologies to predict stress responses. Altogether, this SLR highlights the potential of AI imaging in both biotic and abiotic stress detection to overcome challenges in plant data analysis.

## Introduction

Plant phenotyping involves measuring and studying plant features (i.e., phenotypic traits) such as growth, development, and responses to environmental stimuli and pathogens [1]. Accurate phenotyping is essential to plant breeding and understanding the impact of the environment on plant growth and yield [2]. As the world’s population grows, ensuring adequate and sustainable crop production has become a pressing challenge. In addition, plant stresses adversely affect plant growth, development, and productivity, posing a substantial threat to crop production.

Early detection and accurate diagnosis are essential to manage and prevent plant stress effectively [3]. Advances in both artificial intelligence (AI) and imaging sensor technologies have shown potential for the accurate identification and prediction of plant stress symptoms [4]. The combination of imaging sensors and AI can overcome the limitations of traditional methods like visual inspection, which is time-consuming, expensive, and subjective. Such limitations have spurred the development and adoption of AI, revolutionizing the field of plant stress identification. AI provides a rapid and objective analysis of plant images, enabling early stress identification, even before visible symptoms appear [5]. This empowers farmers to promptly and effectively respond, reducing crop losses and enhancing agricultural productivity [6].

Imaging sensors have shown promise for early stress identification as they image plants quickly, nondestructively, and effectively, recording subtle changes in phenotypic traits [7]. Recently, red, green, and blue (RGB) imaging sensors have become an accessible and pragmatic solution for researchers. The RGB sensor captures the visible spectrum of reflected light and can detect the levels of red, green, and blue light reflected by plants. More informative still, spectral imaging provides detailed information about biochemical and physiological changes induced by abiotic and biotic stressors [8]. By imaging at multiple wavelengths, RGB and spectral sensors identify alterations in plant tissues that are not discernible to the naked eye [9].

Combining AI and imaging sensors has the potential to mitigate the use of pesticides, enhance crop productivity, and bolster worldwide food security by enabling the identification and precise diagnosis of plant stress symptoms [10]. The successful implementation of AI and imaging sensor technologies is contingent upon overcoming obstacles, such as creating precise and resilient AI algorithms capable of managing the intricate and diverse nature of plant images effectively [10].

Although recent progress in AI and imaging sensors has made strides toward identifying plant stress, AI continues to face a range of prevalent challenges. For instance, the fluctuating environmental conditions alongside varying plant reactions pose difficulty for accurately diagnosing and categorizing symptoms of strain or harm [11]. Another core issue is the noticeable lack in diverse, top-tier datasets required for AI model training and assessment tasks. Furthermore, high-end imaging sensors like hyperspectral cameras prove hard to deploy widely due not only to their high costs but also technological sophistication—especially within settings with limited resources [12]. Further challenges are also related to RGB sensors, as while they are more accessible and simple, their relatively sparse spectral data proves inadequate for an exhaustive analysis into physiological complications arising from stresses on plants [12]. Hence, such challenges underscore the need for continued research and development to optimize sensor technologies and AI algorithms, making them more accessible, reliable, and effective for diverse agricultural applications.

Here, we present a systematic literature review (SLR) that provides a comprehensive overview of the current trends and advancements in AI and imaging sensor technologies. SLRs are an established and method to identify, compare, and summarize the findings of studies that address a specific problem, emerging as a very popular tool in computer science [13]. SLRs follow rigorous and well-defined guidelines, enabling researchers to systematically collect papers on a defined topic, consolidating the provided information and identifying further research opportunities. Thus, SLRs present an extra accuracy when comparing to typical literature reviews [14]. Our SLR examines the trends in imaging sensors employed for studying plant stress, the AI algorithms used, and the datasets created to evaluate the effectiveness of these systems. Our review examines the constraints and difficulties associated with these techniques and underscores potential avenues for further investigation. Firstly, we established our research questions that form the basis of the entire SLR: (a) What are the current trends in imaging sensors and AI? (b) What limitations exist to detecting plant stress? and (c) Which AI algorithms have been used to categorize stress symptoms? Secondly, we conducted a scoping review using Google Scholar, searching for relevant papers using general keywords such as “plant stress”, “plant imaging”, and “AI”. Thirdly, we used the results from our scoping review to develop programmable bots that exhaustively search 4 databases, namely Springer, ScienceDirect, PubMed and Web of Science, to find the most relevant studies using these search terms. Fourth, we used OneSearch and programmable bots to access all 4 databases and search for new specialized keywords, including “RGB imaging”, “hyperspectral imaging”, “supervised learning”, etc. Finally, we manually reviewed each output paper with AI assistance using ASReview and placed them into a Zotero cloud database. Altogether, this SLR pinpoints current drawbacks in plant imaging sensors using AI and offers valuable insights about recent advancements in identifying plant stress symptoms.

## AI and Imaging Sensors

Recent advances in AI have been attributed to the rapid development of machine learning (ML) and deep learning (DL) algorithms and improvements to data processing and computational capabilities [15]. Consequently, AI has been implemented in various research fields, including identifying and controlling plant stresses. A key factor responsible for the surge in AI research is the recent availability of copious amounts of high-quality data on stress symptoms provided by modern plant imaging sensors, which AI algorithms can use. In this section, we will delve into the role of imaging sensors and AI in identifying plant stress symptoms. Specifically, we will explore the synergy between imaging sensors and AI, how imaging sensors enable the collection of precise and comprehensive data, and how AI algorithms analyze data and make accurate predictions.

### What are the current trends and limitations for imaging sensors?

For electronic devices to process captured light, the light must be converted into an electronic signal using an imaging sensor [16]. Imaging sensors are commonplace and are found in various electronic devices, such as smartphones and digital cameras, to capture images and videos. The development of imaging sensors has undergone a gradual evolution over time. The RGB, or color sensor, is a device used to capture digital images in the visible spectrum (400 to 600 nm). It contains 3 primary color channels, red, green, and blue, which, when combined, create a colored 2-dimensional image. The RGB sensor is commonly used in remote sensing for various purposes, such as monitoring alterations in the color or texture of plant leaves [17]. RGB sensors are chosen for their high resolution and color depth, which is ideal for applications where detailed visual analysis of growth, plant coloration, and morphometry is crucial. Thus, RGB imaging is the most popular sensor among the research community. Their cost-effectiveness and versatility in both controlled (glasshouses and growth chambers) and field environments make them a popular choice for studies where budget and adaptability are key considerations. Moreover, the adaptability of RGB sensors to diverse crop types and environmental conditions makes them the most versatile tool in agricultural settings.

To analyze plant images and extract features (e.g., phenotypic traits such as color information and plant shape), researchers use image processing methods. Image processing requires the examination of patterns and variations present within an image (edges, lines, or patches) to detect modifications in plant structure [18]. For example, color-based algorithms are commonly used to identify alterations in leaf hues, saturation, or luminance associated with stresses [19]. In addition, texture analysis is conducted on RGB images to detect discrepancies in the leaf texture, which may serve as an indicator of the existence of stress [20]. Integrating color and texture analysis presents a cost-effective and nonintrusive approach to identifying plant stress symptoms. However, despite their widespread use, RGB sensors have several limitations that restrict their applicability. Abiotic changes in leaf color or texture may not be readily apparent for image processing algorithms [12]. Moreover, RGB sensors are also constrained by external factors such as lighting conditions, shadows, and reflections that can affect these sensors” precision [21].

To overcome the limitations of RGB sensors, spectral imaging sensors have become a preferred choice for many researchers because they acquire and evaluate data across multiple narrow spectral bands, providing improved insight into a plant’s physiology [22]. Spectral imaging examines the plant’s spectral signature, enabling the identification of chemical and biological properties that are not discernible using RGB sensors. Spectral sensors capture images using wavelengths that operate beyond the visible spectrum (300 to 900 nm) with a high spectral resolution for every pixel present in the image, thus offering a comprehensive overview throughout the complete electromagnetic spectrum of the plant [23]. By examining the plant’s spectral profile, spectral sensors can detect changes in health indicators, such as chlorophyll concentration or water content, which are not discernible with RGB sensors. This type of information has the potential to identify stresses during their initial phases and monitor their progression over time [24]. The choice of spectral imaging often hinges on the need for in-depth analysis despite the higher cost and data processing requirements. Thus, spectral sensors are particularly beneficial for in-depth analysis of specific crops that exhibit subtle physiological changes under stress. However, spectral imaging is less vulnerable to external variations in illumination and reflections [25] because data is acquired at specific wavelengths less vulnerable to these factors [21]. Despite spectral sensors’ advantages, this type of imaging is more expensive, particularly sensors with a high spectral resolution (900 to 2,500 nm) [21]. Additionally, spectral imaging generates a substantial volume of data requiring specialized software for processing and analysis. Even with specialized software, the process can be laborious and requires expert knowledge in data analysis [8]. Challenges such as these are often why researchers opt for RGB sensors, namely their cost-effectiveness, simpler operational requirements, and compatibility with commonly available devices such as smartphones and digital cameras. Nevertheless, spectral imaging is a powerful technology for studying plant stress responses, particularly when combined with AI algorithms and ground-truth data. Thus, the choice of spectral imaging often revolves around the need for in-depth analysis regardless their higher cost and data processing requirements.

Despite the advancements in spectral imaging, researchers occasionally turn to more specialized sensors tailored to specific experimental requirements. Among these, fluorescence imaging stands out as a unique and powerful imaging sensor. This sensor excels in detecting subtle changes in plants’ structural and biochemical attributes at specific wavelengths (250 to 700 nm), making it particularly effective for identifying stress responses [8]. Researchers leverage fluorescence imaging to differentiate between stressed and unstressed plants based on variations in their fluorescence emissions, which can be translated into photosynthetic responses [26]. However, a notable consideration in fluorescence imaging is the need for dark adaptation. Plants often require a period of acclimation in darkness to ensure accurate fluorescence measurements, as ambient light can influence fluorescence signals [27]. This requirement can add time and complexity to the experimental setup, limiting the method’s convenience and applicability in some research contexts. Fluorescence imaging is especially relevant in controlled environments where conditions for dark adaptation can be managed, despite its higher cost and specialized operational requirements [27]. Although its effectiveness in revealing stress-induced alterations in plant physiology, fluorescence imaging is not as widely employed as RGB- or spectral-based sensors. Hence, fluorescence imaging under utilization stems from several factors, including its high cost, which limits its accessibility and restricts its use primarily to well-funded research projects. In addition, the specificity of fluorescent sensors means they are only applicable in certain contexts, particularly where the extraction of specific fluorescent features is crucial. As a result, the use of fluorescence imaging in plant stress detection, while highly effective in specific scenarios, remains less common than RGB due to these practical, financial, and procedural constraints.

Such can also be said for the use of thermal sensors. Thermal imaging or infrared sensors are devices that detect and measure infrared radiation emitted by objects or surfaces to create images based on temperature variations [28]. Unlike visible light, which is detected by traditional RGB sensors, thermal imaging sensors operate in the infrared spectrum (7 to 14 μm) and are used to monitor plant stress by measuring the infrared radiation emitted by plants [29]. These sensors offer a unique perspective by allowing researchers to detect variations in plant temperatures, which can indicate water stress, disease, or other physiological changes not visible to the naked eye. Thermal imaging is particularly valuable in large-scale agricultural monitoring of temperature-related stress, where early detection of stress factors can lead to timely intervention and improved crop management [30]. Despite its advantages, thermal imaging is not as frequently used as RGB or spectral sensors in plant stress research. One reason for this is the complexity and cost associated with thermal imaging equipment. High-quality thermal sensors tend to be expensive, making them less accessible. Moreover, thermal imaging requires specific expertise to accurately interpret the data, as temperature readings can be influenced by environmental conditions such as humidity and wind [31]. This added complexity means that thermal imaging is not always the most practical choice for researchers, particularly in field tr where controlling environmental variables is challenging [32]. Furthermore, thermal imaging, while effective in detecting temperature-related stress indicators, may not provide the comprehensive data needed for a complete understanding of plant health. RGB and spectral sensors, with their ability to capture a wider range of data including color and spectral reflectance, offer a more holistic view of plant conditions. These sensors can detect subtle changes in color and reflectance that are indicative of a variety of stress factors, including nutrient deficiencies, pest infestations, and disease presence, which are not always discernible through thermal imaging. Consequently, many researchers opt for RGB or spectral imaging sensors as they provide a more versatile and detailed analysis of plant health, suitable for a broader range of research applications.

Similar observations can be made regarding the use of satellite imaging in plant stress research. Satellite imagery, captured from orbiting satellites fitted with a variety of sensors such as RGB, and multispectral and thermal features provide an unparalleled perspective for researchers to observe broad environmental changes and evaluate plant health over large areas [33]. Satellite imagery is particularly instrumental in documenting events like drought, deforestation, or pest infestations, providing key data that aids ecological conservation efforts along with the management of agricultural practices at macro level [34]. Despite these benefits, satellite imaging is still in its infancy for detailed plant stress research, especially when compared to aerial and ground-based RGB or spectral sensors. A primary limitation of satellite imaging is its spatial resolution [35]. While recent advancements have substantially improved the resolution of satellite images, they often cannot match the fine detail captured by ground-based sensors [35]. An additional challenge with the use of satellite imagery is the frequency of data acquisition [36]. Satellite overpasses may not always align with the specific timing needed for certain research studies, leading to gaps in data collection. This temporal limitation can be critical in plant stress research, where changes can occur rapidly and need to be monitored very frequently for an accurate analysis. Furthermore, satellite imagery can be affected by atmospheric conditions such as cloud cover, which can obstruct clear views of Earth’s surface [36]. This issue adds an element of unpredictability, limiting the reliability of satellite data for continuous monitoring purposes. As a result, while satellite imaging offers invaluable insights for large-scale environmental monitoring, it is often complemented by or secondary to the more detailed and frequent data provided by RGB or spectral imaging in the context of focused plant stress research.

LiDAR (Light Detection and Ranging) is another type of imaging sensor that is increasingly used to monitor plant stress. LiDAR sensors work by firing pulses of laser light at an object and record the time it takes for a reflection to return, building a detailed 3-dimensional (3D) point cloud about the shape and structure of objects. LiDAR is particularly skilful at capturing the structural properties of plants, such as canopy height and leaf area index, informing the presence of stressors like drought or disease [37]. Recent research has shown that structural irregularities in plants can be successfully revealed by point cloud data from LiDAR imaging sensors, offering early indicators of stress before they are even noticeable using conventional imaging techniques [38]. Despite such advantages, LiDAR use is not yet very frequent commonly for several reasons. Firstly, the cost of LiDAR equipment, especially high-precision models, can be high, making it less accessible for many research projects. Secondly, while LiDAR excels in capturing structural data, it does not provide information on the color or spectral properties of plants, which are crucial for identifying a wide range of stress symptoms. Thirdly, the operation and data processing of LiDAR systems can be complex [39], as its interpretation requires specialized software and expertise in 3D data analysis. Finally, LiDAR complexity can be a deterrent for researchers who require straightforward and quick data analysis methods, especially in large-scale studies where efficiency is key. While LiDAR offers unique insights into the physical structure of plants and vegetation, its application in plant stress research is often limited to specific studies where detailed structural information is paramount. These studies are frequently supplemented by the more comprehensive data provided by RGB or spectral imaging technologies. However, the future may see more integration of LiDAR data with AI algorithms to enhance its effectiveness in detecting and analyzing plant stress, particularly in areas where structural changes are critical indicators.

Finally, in recent years, the integration of data from various imaging sensors through multimodal approaches has emerged as a promising strategy for comprehensive plant stress analysis [40]. Multimodal approaches combine aspects from RGB, spectral, and other imaging sensors to capture a more complete image representation of a plants health condition. By using a multitude of imaging sensors, researchers will have a greater variety of features (phenotypic traits) to select from, enhancing analysis processes and encouraging researchers to focus on using hybrid approaches to tackle multimodal datasets. Nevertheless, many challenges exist with multimodal approaches as they can be challenging due to varying complexities related with data incorporation alongside demands for sophisticated algorithms capable of effectively analyzing bundled datasets, which may greatly increase computational requirements. Thus, while offering more rich analysis, multimodal necessitates cautious evaluation regarding different sensors’ format compatibility along-with their synchronisation.

### What are the current trends and limitations of AI?

AI refers to programs that simulate human intelligence, enabling machines to perform reasoning, learning, perceiving, and decision-making [41]. AI has several subdisciplines, such as ML, DL, natural language processing, and computer vision. AI algorithms are used to analyze extensive datasets and detect patterns that can be used to develop predictions. AI’s capability to process and analyze data from various imaging sensors is critical in plant stress detection. Advanced AI algorithms, particularly in ML and DL, are designed to adapt to the inherent variability in sensor data. To mitigate the challenges posed by sensor variability, standardized preprocessing techniques are employed. These techniques, such as normalization, calibration, and noise reduction, are crucial for preparing sensor data for AI analysis. They help in reducing the discrepancies between datasets, allowing AI models to focus on relevant plant stress indicators. For instance, DL models can be trained to recognize and adjust for variations in lighting or atmospheric conditions that affect image quality, ensuring consistent analysis across different datasets. Among the many subdisciplines of AI, ML is currently one of the most popular and frequently used. AI learning processes are categorized into either supervised or unsupervised learning. In supervised learning, algorithms learn from labeled examples in a dataset. These examples include input data, such as plant images or spectral measurements, and corresponding labels indicating whether the imaged plant displays signs of tolerance or sensitivity to specific stress [42]. Supervised learning algorithms analyze these labeled examples to identify patterns between input data and stress, e.g., plant height changes in response to either control or drought stress conditions. Supervised learning then generalizes this knowledge allowing it to make predictions on unseen data. On the other hand, unsupervised learning involves exploring unlabeled datasets where the computer has no information on whether the data relating to a specific plant trait was collected under stress or control conditions. Unsupervised algorithms focus on identifying inherent structures, recognizing patterns, or similarities within the data, aiming to cluster similar features together [42]. This allows for discovering relationships between features based solely on the characteristics of the data. Unsupervised learning can identify hidden patterns in plant stress datasets and provide valuable insights into the structure and diversity of stress responses [43]. Both supervised and unsupervised learning are critical AI principles and often dictate what algorithms a researcher can or cannot use for their analysis.

ML allows computers to improve performance on a given task by learning without requiring explicit programming [44]. It involves feeding algorithms (such as the support vector machine [SVM] or artificial neural networks [ANN]) with data and allowing them to learn and improve from experience, making it a dynamic and adaptable AI subdiscipline. Being the most popular subdiscipline in AI, ML has seen a surge in use due to the increased availability of large datasets, enhanced capabilities of computers, and the emergence of novel algorithms [45]. Imaging sensors produce large datasets in a very short time, promoting the use of AI [5]. Moreover, ML has revolutionized the process of data analysis in plant imaging, primarily due to the emergence of increasingly sophisticated algorithms. Images of healthy and stressed plants can facilitate the training of ML algorithms providing the ability to identify stress-related symptoms accurately. ML also provides the capability to analyze data in real time, providing researchers with timely information on the health of their crops. Generally, the use of ML for plant stress detection often involves researchers working with user-friendly software applications that are designed for nonexperts. These applications allow researchers to easily upload their imaging data and have ML models analyze their images (for stress symptoms). An example of such user-friendly software includes the popular PlantCV [46] library, which makes analyzing RGB and spectral imaging data accessible even for researcher without a background in computer science and AI. However, despite the advantages of ML, these algorithms require large training datasets before they are capable of making accurate and reliable predictions. If the training data is insufficient, the predictions generated by ML may be inaccurate [47], which may lead to erroneous stress classification [48]. Furthermore, ML algorithms are also sensitive to input variation, including changes in image quality and lighting conditions, leading to reduced accuracy and reliability.

DL is a subdiscipline of ML that uses deep neural networks (DNNs) to tackle complex problems. DNNs are ANNs that have a complex arrangement of interconnected nodes [49]. The architecture of a DNN is inspired by the structure and function of the human brain, allowing them to emulate the ability to process and analyze information [50]. DNNs are structured hierarchically into layers. Each layer is tasked with acquiring distinct features (phenotypic traits) and allows for various types of input data (e.g., numerical, textual, or visual). DNNs can acquire intricate data patterns, rendering them appropriate for processing complex datasets [51]. Feature extraction, i.e., taking out from a complex plant image a phenotypic trait such as canopy architecture, is a commonly encountered challenge in ML as it cannot extract these features automatically. This often requires researchers to preprocess their data before analysis. To overcome this challenge, DL facilitates the autonomous extraction of features from plant images by algorithms, enhancing the efficiency and precision of collecting phenotypic traits [52]. Also, unlike ML models, researchers lack the optional user-friendly software to streamline their analysis process. This is because DL models require more computational resources for training and often need some technical understanding to tweak the algorithms to their imaging data. To address these challenges researchers often engage in interdisciplinary collaborations, bringing together expertise from computer science to handle the technical aspects of DL. This approach allows for a more effective application of DL in plant stress research, leveraging the strengths of various disciplines to advance the field.

In plant imaging, convolutional neural networks (CNNs) have been used to estimate and classify stress-related symptoms (e.g., lighter color in field plots). As previously mentioned, ML struggles to extract features from background noise in images [53], whereas CNNs can bypass the complex nature of feature extraction. Generative adversarial networks have also become an alternative DL method to produce synthetic images of plants, enabling researchers to artificially increase the size of their datasets and improve the efficiency of their prediction and classification models [54]. Also, AI models are increasingly being developed to integrate data from multiple sensor types, such as combining RGB, thermal, and spectral data. This integration allows for a more comprehensive analysis of plant stress, leveraging the strengths of each sensor type and compensating for their individual limitations. Despite the exciting prospects of DL, there are still several limitations to its use. DL algorithms require substantial amounts of data of superior quality, containing both stress and stress-free images, which is laborious and expensive to acquire. DL also demands substantial computational resources requiring specialized hardware, namely powerful graphics processing units to train on large datasets as well as experts in computer science. In fact, DL models may struggle when generalizing predictions when presented with features (phenotypic traits) different from the ones presented in the training dataset [55]. However, the robustness of AI models to handle sensor variability is continuously being enhanced. Techniques like data augmentation and transfer learning are employed to expose models to a wide range of conditions, thereby improving their ability to generalize across different sensor data. This is particularly important in plant stress detection where environmental factors can substantially influence sensor readings. Despite these challenges, continued advancements in AI can address these limitations and further improve ML and DL algorithms for plant stress identification [48].

Furthermore, for AI algorithms in plant stress detection, the performance of algorithms is considerably influenced by the quality of training data. Effective AI models rely on diverse, large, and representative datasets; limited or biased data hinder generalization across different plant species and stress conditions. Ensuring dataset reproducibility is essential for the reliability and applicability of AI models. Standardized data collection and preprocessing methods are vital to maintain dataset representativeness and reproducibility, addressing data noise, missing values, and dataset balancing. Cross-study comparability, real-world applicability, dataset accessibility, and ethical considerations are critical in advancing AI applications in plant phenomics. Additionally, AI algorithms are designed to be flexible and robust, adapting to diverse agricultural settings and crop types. They can manage variations in sensor data across environmental conditions, enhancing the precision of AI in various agricultural applications. This adaptability allows AI models to recognize and adjust for factors such as soil type, climate, and crop variety, thus enhancing AI’s utility in detecting plant stress and providing insights for sustainable farming practices.

## SLR Methodology

To establish the modus operandi of imaging sensors and AI in plant phenotyping, we conducted a SLR following the Preferred Reporting Items for Systematic Reviews and Meta-Analyses guidelines [14]. We designed an exhaustive investigation strategy that incorporated a 3-phase process to search, collect, and analyze the published results in plant imaging and AI from selected journal repositories. SLR limitations include potential omissions as gray literature such as graduate studies or conference papers and selections bias to the researchers’ inclusion and exclusion criteria and being confined to English published work. Although the SLR system is known to have limitations such as the absence of relevant details and repeatability of the results, SLRs have become a well-established method to consolidate knowledge of existing empirical studies before conducting a new one [13].

### Research questions

To conduct our thorough literature search, we first established a series of research questions to pinpoint the information sought through the SLR specifically. The following research questions also establish the foundation for the SLR and its subsequent acquisition of relevant papers:

1. What is the present status of research concerning the use of imaging sensors and AI for identifying plant stress symptoms, and how has this particular domain progressed since 2006?
2. Which AI algorithms can analyze data obtained from imaging sensors to identify and categorize plant stresses?
3. What limitations exist in current AI and imaging-sensor technologies for identifying plant stress symptoms, and what strategies can be implemented to improve their effectiveness?

### Review search strategy

**Fig. 1.**
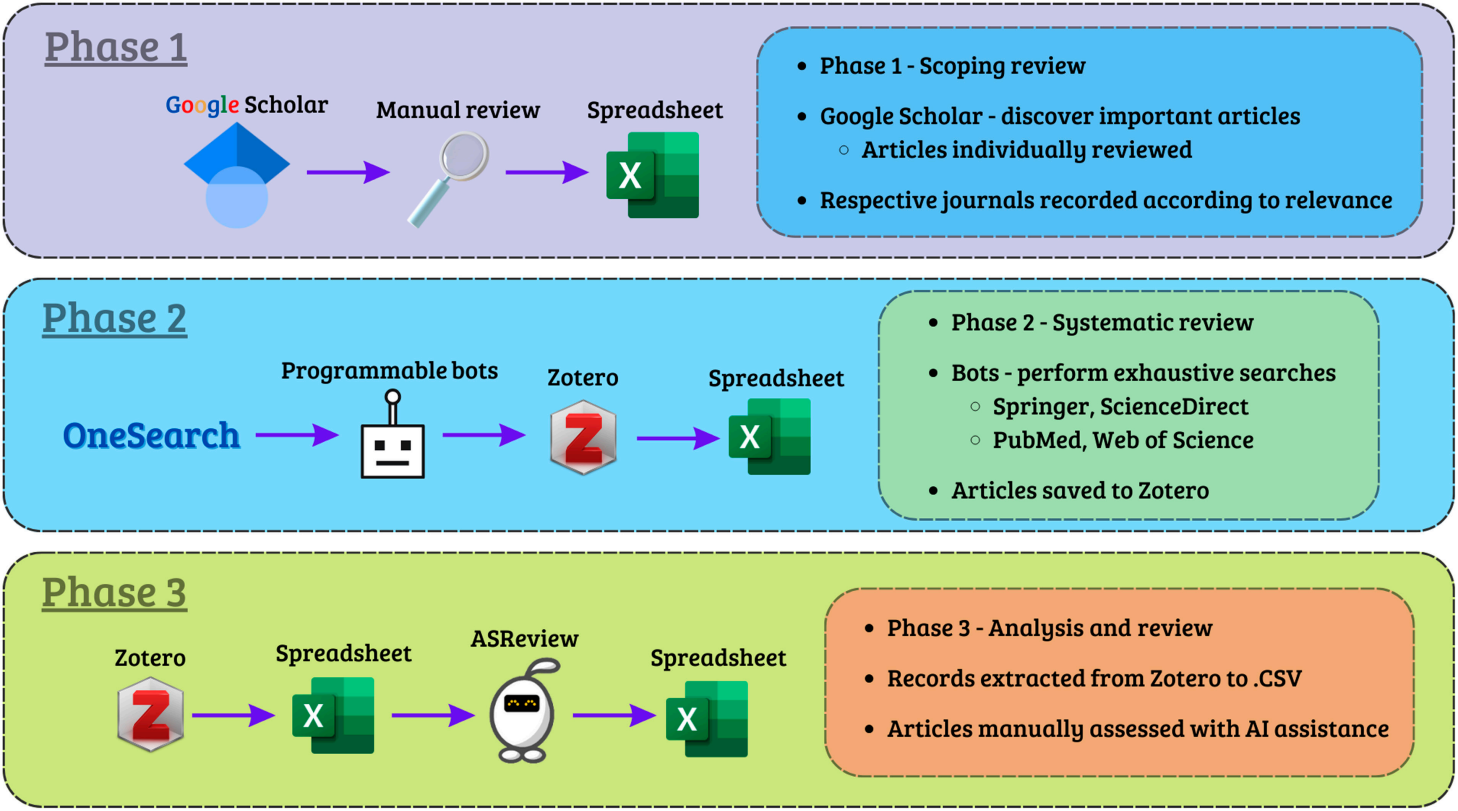
Overview of the 3-phase search strategy used in this SLR. Phase 1 focuses on an initial scoping review. Phase 2 focuses on the systematic review and capture of the resulting studies. Finally, Phase 3 focuses on the analysis and review of the primary study cohort.

The Preferred Reporting Items for Systematic Reviews and Meta-Analyses guidelines are a crucial component of every SLR, providing a framework for conducting the review process [14]. This SLR comprises a 3-stage methodology (Fig. 1) to procure relevant studies from 4 journal repositories (hereafter referred to as databases). Each phase aims to extract relevant research papers (hereafter referred to as studies) broken down into information for subsequent phases. The knowledge acquired from prior phases is applied in successive iterations, resulting in the discovery of more relevant studies in later phases. Our SLR approach enables an efficient and analytical review by guaranteeing comprehensive and exhaustive scrutiny of each database. Phase 1 is an initial scoping review that uses influential keywords to gather information. Phase 2 is a complete search procedure that uses automated bots to systematically explore specialized keywords and create a string of specialized information. The final phase, Phase 3, is an analysis review of all studies identified during Phase 2. Further details of our SLR strategy are outlined below in subsections.

#### Phase 1 - Scoping review

The first phase of our SLR was a scoping review using the academic search engine Google Scholar [56]. This scoping review aimed to investigate image-based phenotyping used in plant stress and determine which databases contained the most relevant studies. We used a predetermined set of general keywords to search Google Scholar, which was kept general and covered the areas of plant stress, AI and imaging technologies. For example, we used “biotic stress” combined with “machine learning”, creating a single search string in Google Scholar. The goal of Phase 1 was to determine the best-performing keywords to use in Phase 2. Scholarly articles were retrieved from Google Scholar in Portable Document Format (PDF) and systematically archived in an external database. The outcomes of each search were duly documented in a spreadsheet to retain a record of all the studies from Phase 1. In a nutshell, Phase 1 reviews the: (a) keywords provided via the publication, (b) the corresponding database, (c) the plant imaging sensor used, and (d) AI algorithms listed in the study. Our scoping review selected 4 databases (Springer, ScienceDirect, PubMed, and Web of Science), as well as the inclusion and exclusion criteria for Phase 2. In addition, Phase 1 generated a new compilation of specialized keywords such as “hyperspectral imaging”, “fluorescence imaging”, and “support vector machines”, which were subsequently used in Phase 2.

#### Phase 2 - Systematic review using programmable bots

Phase 2 makes up the core of this SLR and uses OneSearch as its key search engine. OneSearch is an established academic database that enables further minutia in the form of search string combinations. In general, SLR requires manual exhaustive searches across multiple databases. To streamline the search process and reduce human error in database searches, we developed programmable bots using the open-source Python library PyAutoGUI [57]. Our automated bots system continuously retrieved search results in a 2-step process to obtain the total count of research studies discovered after each search and to document each study in a Zotero database [58].

Up to date, automation of the repetitive SLR tasks using AI has been an object of ongoing research as reviewed by [59], yet to our knowledge, this SLR is the first to present programmable bots capable of autonomously conducting an entire search process with great minutia. The bots were created using Python as the programming language because it is easy to use and contains many user-created libraries (all code used in this manuscript is freely available on GitHub at https://github.com/Walshj73/data-processing-bot.git. We used the Python library PyAutoGUI to automate basic tasks and control the mouse and keyboard systematically. Each bot was implemented in a Python script executed using the computer’s terminal. Each Python script contained a single class we termed “bot”. This “bot” uses a specific location to execute its commands using a pixel-based format on the computer monitor. Hence, the “bot” operation depends on the display resolution of the host computer. For this SLR, we used a computer display with 1,920 × 1,080 standard resolution. However, our search bots can work with any pixel resolution. Users must provide the bots with the x and y pixel coordinates of the object they intend to interact with. Once the bot understands the location of the objects, it can then begin performing its instructions. Our scripts contain 3 loops causing the entire script to rerun once it reaches a certain point. Specialized keywords in Phase 2 are split into 3 groups: those associated with plant stress, imaging technologies, and AI. Each group would have a single loop in a bots script and iterate for the total keywords in that grouping. Because the bots perform many tasks uninterruptedly, the risk of error increases with time. To overcome this risk, our bots have error-checking capabilities as a “checkpoint system”, allowing the bot to revert to a previous save state before an error occurs. The “checkpoint system” provides the bots with independence and enables them to work extensively without human interference.

Our bots use OneSearch and a specialized keyword dataset. Nevertheless, researchers can modify the code to accommodate other database search engines such as Google Scholar. This can be achieved by providing the bots with fresh pixel coordinates and an updated list of search instructions. In addition, we developed our scripts using open-source software. Hence, our bot scripts can be easily customised for other research endeavours. For example, a researcher interested in molecular biology and the use of gene editing in any organism can edit our programmable bots scripts with ease and create their own SLR.

Programmable bots carry out simple sets of instructions. In our case, these instructions are focused on performing searches using OneSearch. A search can yield one of 3 possible outcomes: (a) positive results with relevant studies found, leading to the bot documenting the outcome, specifically the number of studies discovered, onto a spreadsheet and archiving all studies onto the Zotero database; (b) search was successful, but no studies were found, leading to the bot recording the outcome (i.e., the numerical value of zero) onto the resulting spreadsheet. As no studies were found, the bot will proceed with the next search; and (c) unsuccessful search due to the occurrence of an error. Here, the bot would use its “checkpoint system” to revert to an earlier saved state in memory, enabling it to rerun the previous task. When multiple errors are encountered, human assistance is required to restart the search process.

Using the specialized keyword dataset, our bots meticulously constructed keyword combinations which enabled the construction of precise search strings. We began with a single keyword focusing on plant stress, termed “root keys” (e.g., abiotic stress), that acted as the first combination term. In the initial stage of Phase 2, we used 6 “root keys” and 4 databases, which resulted in 24 queries. Next, the bots retrieved keywords directly linked to the root keys focusing on plant imaging sensors, termed “parent keys” (e.g., RGB imaging sensors), which we combined with “root keys” using operators such as “AND” and “OR”. Bots performed 2,016 search queries by concatenating 6 root and 56 “parent keys”. Finally, focusing on AI, we created the term “child keys” (e.g., supervised learning) to connect to the “parent keys directly” and subconnect to the “root keys”. The “child keys” are the final layer of our SLR search process. In the end, our programmable bots combined 6 “root keys”, 56 “parent keys”, and 14 “child keys” in 4 databases, resulting in 28,224 searches and greatly simplifying the analysis of Phase 3.

#### Phase 3 - Analysis and review

In Phase 3, we first analyzed the results from Phase 2 regarding duplicates and used Zotero to remove any duplicate studies automatically. The remaining studies were then exported in a comma-separated values (CSV) file format and saved to a local storage device. Next, the AI-powered SLR tool, ASReview [60], was used to import each CSV file for evaluation. The use of ASReview enabled the review process to be streamlined, reducing the time required to evaluate each study. ASReview was developed specifically for use with SLRs and includes intelligent analytics with ML algorithms to assist researchers in evaluating their results. We used the default algorithms and parameters in ASReview to screen each study for relevance. The ASReview software ordered the studies from most to least relevant. We then performed a manual review of the studies based on the inclusion/exclusion criteria defined below. The final dataset was re-exported to a new CSV file where we performed a strict evaluation of each study based on the data used (i.e., use of imaging sensors), the methods employed (i.e., type of AI), and the results generated (i.e., algorithms performance).

### Inclusion and exclusion criteria

To achieve a first-rate SLR, we must determine if a study is relevant. We evaluated all the studies compiled in Phase 3 using a predetermined set of criteria, including document type (e.g., journal article and conference proceeding), publication year, and content type. We categorized studies for inclusion (acceptable) and exclusion (unacceptable) as below:

Inclusion criteria

• The study has been published between 2006 and 2022 inclusive.
• The study identifies plant stress, classification, quantification, and prediction, including pest-related damage.
• The study uses an imaging sensor to analyze, including RGB, spectral, etc.
• The study includes some form of AI, such as ML or DL algorithms and supervised and unsupervised methods.

Exclusion criteria

• The study was published before 2006 or after 2023. New studies published at the beginning of 2023 were not considered for selection and were automatically removed.
• The study does not focus on the area of plant stress.
• The study does not use an imaging sensor or imaging-related datasets and instead relies on handheld sensors.
• The study does not use AI and only focuses on statistical methods.
• The study is a case study or article review paper.

Furthermore, we included some minor criteria using OneSearch’s built-in configurations to facilitate the filtering of the studies, namely:

OneSearch configurations

• The study is either a book chapter, conference proceeding, or journal article.
• The study is written in English.
• The studies retrieve keywords from “full text” and not only from a “summary or abstract”.

## Results and Discussion

### The use of programmable bots increased the number of relevant studies

**Fig. 2.**
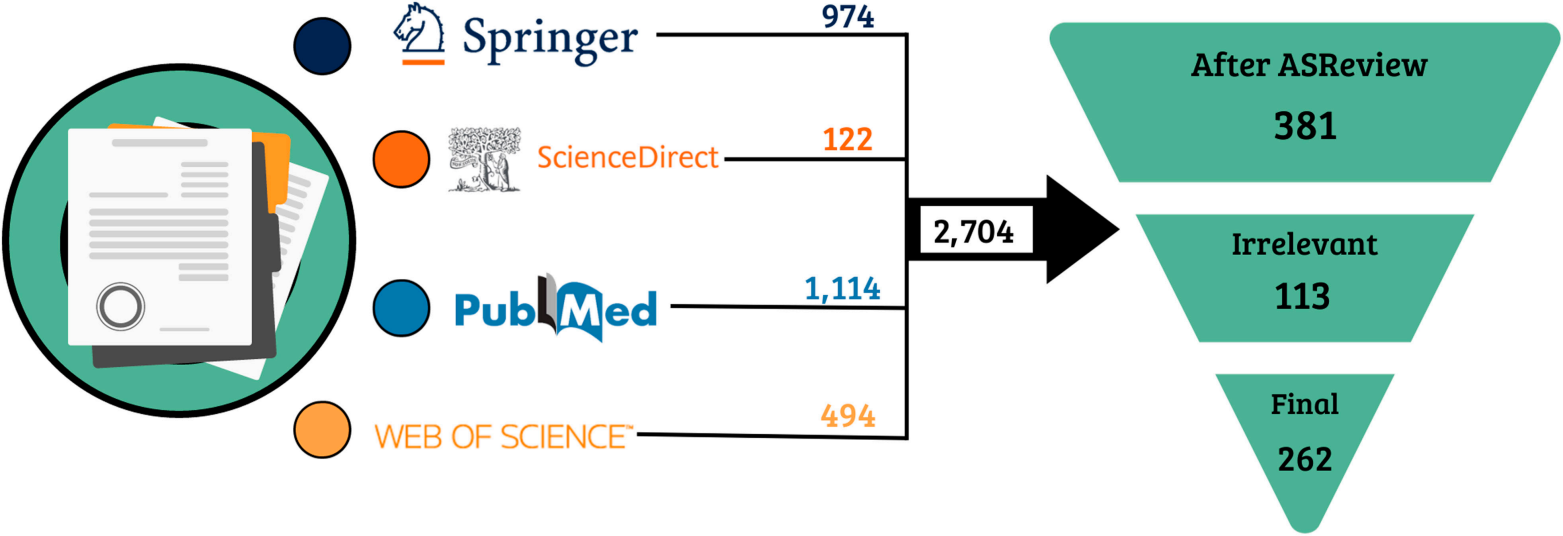
Summary of the total number of studies retrieved by our SLR, which targeted AI and imaging sensors investigating plant stress. Four databases were used to search relevant studies using manual inspection and programmable bots. Retrieved numbers from each database are presented: Springer in dark blue, ScienceDirect in dark orange, PubMed in light blue and Web of Science in light orange. The results yield 2,704 identified studies, as indicated in the bold dark arrow. The final results are presented in the inverted green pyramid on the right-hand side, with each selection step indicating the number of studies individually assessed with ASReview, and considered irrelevant, generating the final 262 studies examined in this SLR.

This SLR aims to investigate the current trends in imaging sensors and the use of AI to interpret plant stress-related symptoms. Our new SLR strategy with programmable bots resulted in a total of 2,704 studies being retrieved from the 4 selected databases using the string of “root”, “parent”, and “child keys” (Fig. 2). After following 3 stringent phases of data search, we identified 262 studies that substantially contribute toward identifying plant stress symptoms using AI and plant imaging.

We observed a disparity in final results numbers (262) retrieved between the 4 databases, as ScienceDirect and Web of Science found a relatively low number of studies. From the 122 studies found in ScienceDirect, only 33 underwent the analysis process, as 90 were deemed irrelevant. Of the results found using Web of Science, only 30 were deemed relevant. On the other hand, from the Springer database, 974 studies were obtained, with 101 being selected for final analysis. ScienceDirect’s retrieval rate was subpar compared to the other 3 databases (12%), as PubMed found 37% and Springer 38%. The low number in ScienceDirect might be attributed to the database’s content, i.e., its journals, that predominantly focus on experimental plant biology or technological aspects of imaging sensors. Consequently, studies focusing on identifying stress-related symptoms using AI and plant imaging are beyond the scope of most ScienceDirect journals. On the other hand, Springer and PubMed have the highest number of final studies retrieved. This result might be explained by the widely recognized “Plant Methods” journal, affiliated with BioMed Central, which serves as a primary platform for many papers published concerning AI and imaging sensors. We observed that Web of Science found highly relevant studies, but 93% of the 494 studies were discarded as duplicates retrieved by one of the other 3 databases.

Our SLR yielded a notably higher number of relevant studies than other SLRs conducted across multiple research fields. Typically, an SLR retrieves less than 1,000 studies, narrowing to less than 100 relevant studies [61–63]. Yet, our SLR successfully identified 262 relevant publications from 2,704 studies, considerably larger than what is typically found through manual review. This outcome can be attributed to our implementation of a 3-phase review strategy and the use of programmable bots, which clearly advanced the efficiency of our search process. As a result, our findings strongly indicate that programmable bots can effectively enhance the number of relevant studies identified, preventing potentially relevant research from being overlooked.

### Greater demand for AI in plant stress analysis since 2020

**Fig. 3.**
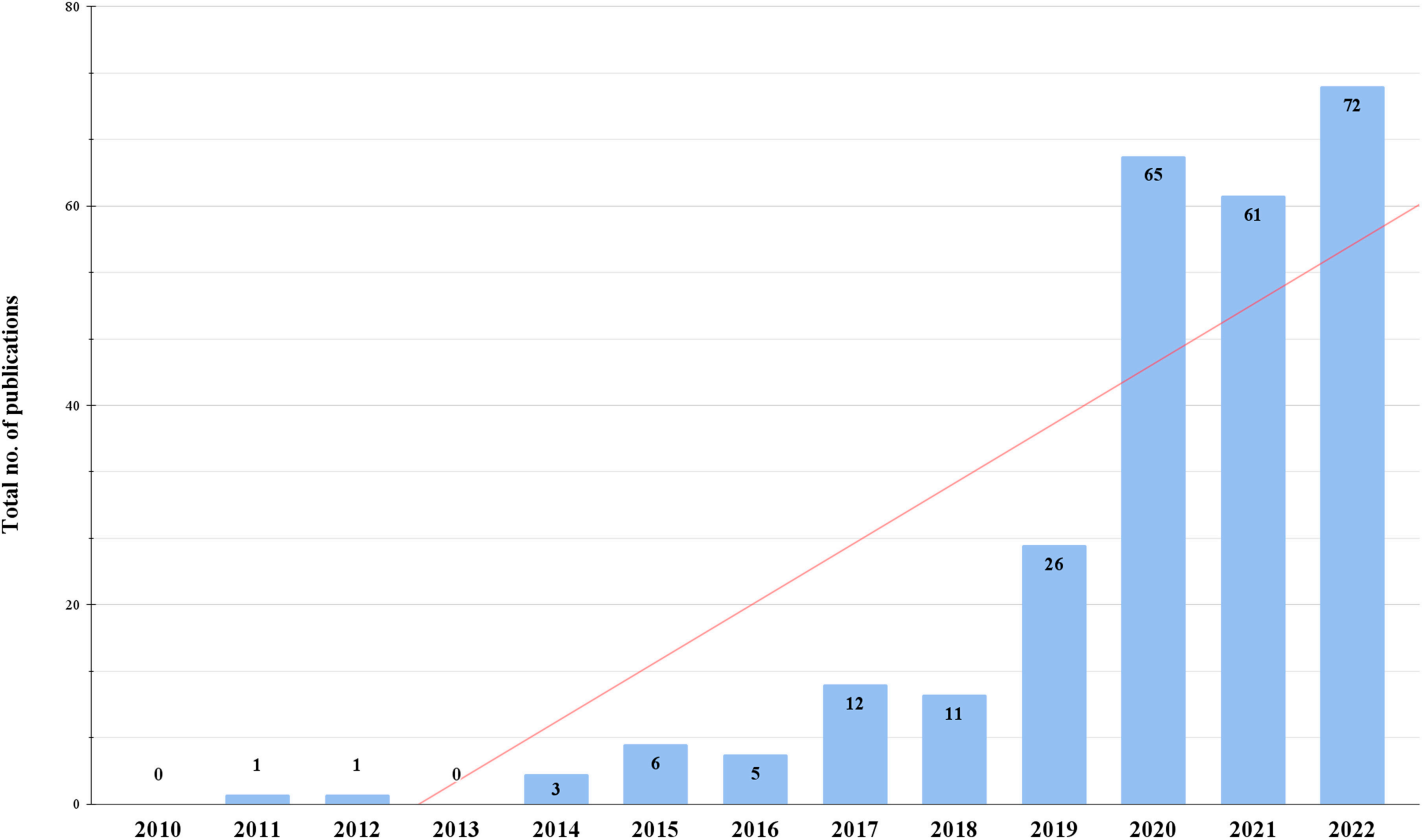
Summary of the total number of studies published by year using AI and imaging sensors to investigate plant stress responses.

Our SLR examined 262 relevant studies published since 2006 (Fig. 3). We can observe a trend of AI emerging in early 2017, with a noticeable surge beginning in 2019, increasing from 26 to 65 studies in 2020 and rising to 72 publications in 2022, leading us to speculate that this trend will persist into 2023 and beyond. We also observed fewer publications in 2020, even though the trend line indicates that 2021 should have more studies. This slight decline from 65 studies in 2020 to 61 in 2021 is most likely due to the COVID-19 pandemic, as many researchers were forced to suspend their work or work remotely.

Interestingly, we observed that no results were found before 2010, possibly because access to imaging sensors was difficult due to their high price and maintenance expenses. Instead, researchers opted for destructive phenotyping. Even though few studies with AI and imaging sensors were published in 2011 and 2012, considerable advances were made in the area [8,64]. For example, Mewes et al. [64] used a hyperspectral mapper affixed to an aircraft. They presented a spectral angle mapper (SAM) and an SVM to identify powdery mildew in wheat. The article reflects on the sensor’s spectral range, which enables imaging at both near-infrared and short-wave infrared frequencies, which was a breakthrough at that time [64]. Mahlein et al. [8] also used a SAM to detect *Cercospora* leaf spots, powdery mildew, and leaf rust in beet leaves. They similarly used a hyperspectral sensor but under glasshouse conditions. Although the use of AI and imaging sensors was sparse before 2019, published papers show advances in the area despite cost-related challenges. After 2017, the cost of imaging sensors dropped considerably, allowing more research groups to conduct nondestructive phenotyping and implement AI algorithms. For example, Nagasubramanian et al. [65] tested a deep CNN with a hyperspectral imaging sensor to detect charcoal rot in soybean, using the same Pika XC hyperspectral line sensor previously seen in 2011 [8,64]. This observation suggests that researchers use similar technology in imaging sensors but more modern AI algorithms [64,65]. Taken together, our results (Fig. 3) point to a surge of published papers in 2020 that can be attributed to the affordability of imaging sensors as well as an explosion of AI due to the development of DL techniques and the emergence of large-scale neural networks, leading to a rise in phenotyping studies.

### Open-source datasets are causing a data revolution

Another reason for this surge in published papers since 2019 could be due to publicly available datasets, which have become popular among researchers due to the promotion of open-source publications. We found that approximately one-third of the studies (36.4%) used a publicly available dataset to perform their research. This included datasets from open-source repositories such as Kaggle or Data.gov. Below is a compiled list of some of the most frequent publicly available datasets to appear among the cohort:

• PlantVillage: Publicly available dataset comprising over 50,000 images of healthy and diseased crop leaves categorized across numerous plant species and diseases [66]. It is widely used for developing and testing AI algorithms in plant disease diagnosis and has become a cornerstone resource in agricultural AI research.
• PlantDoc: This dataset contains 2,598 data points in total across 13 plant species and up to 17 classes of diseases [67].
• Rice Leaf Diseases Dataset: The dataset contains 120 .JPG images of disease infected rice leaves. Each image is grouped into 3 classes based on the type of disease with 40 images in each class [68].
• Cassava Leaf Disease Classification: A competition dataset containing 21,367 labeled images of cassava leaf disease collected during a regular survey in Uganda [69].
• Soybean (Large) Dataset: This dataset comprises 19 classes of both healthy and un-healthy soybean images. The dataset has a total of 307 instances and 37 features [70].
• CGIAR Computer Vision for Crop Disease: This publicly available dataset contains 876 images for training and 610 images for testing, which can be used for the identification of wheat rust [71].

The PlantVillage dataset, introduced in 2015, has become one of the most widely used and recognized publicly available datasets. PlantVillage was generated to advance research in mobile disease diagnostics, yet it has been used in many studies to train and evaluate novel AI algorithms [72–74]. For example, one study used a lightweight CNN, whereas another used a pretrained version of the MobileNet-V2 architecture to study biotic stress [75,76]. Most studies that used a publicly available dataset were published recently, suggesting that open-source datasets are a key factor in the publication surge from 2020 onward. These findings are supported by 58 studies using DL which employed the PlantVillage dataset, reinforcing open-source software’s effectiveness in advancing DL-based plant stress research. Ultimately, we can speculate that the limited research before 2015 could be due to the lack of publicly available image datasets.

### Spectral imaging takes center stage and becomes an emerging choice for plant imaging

**Fig. 4.**
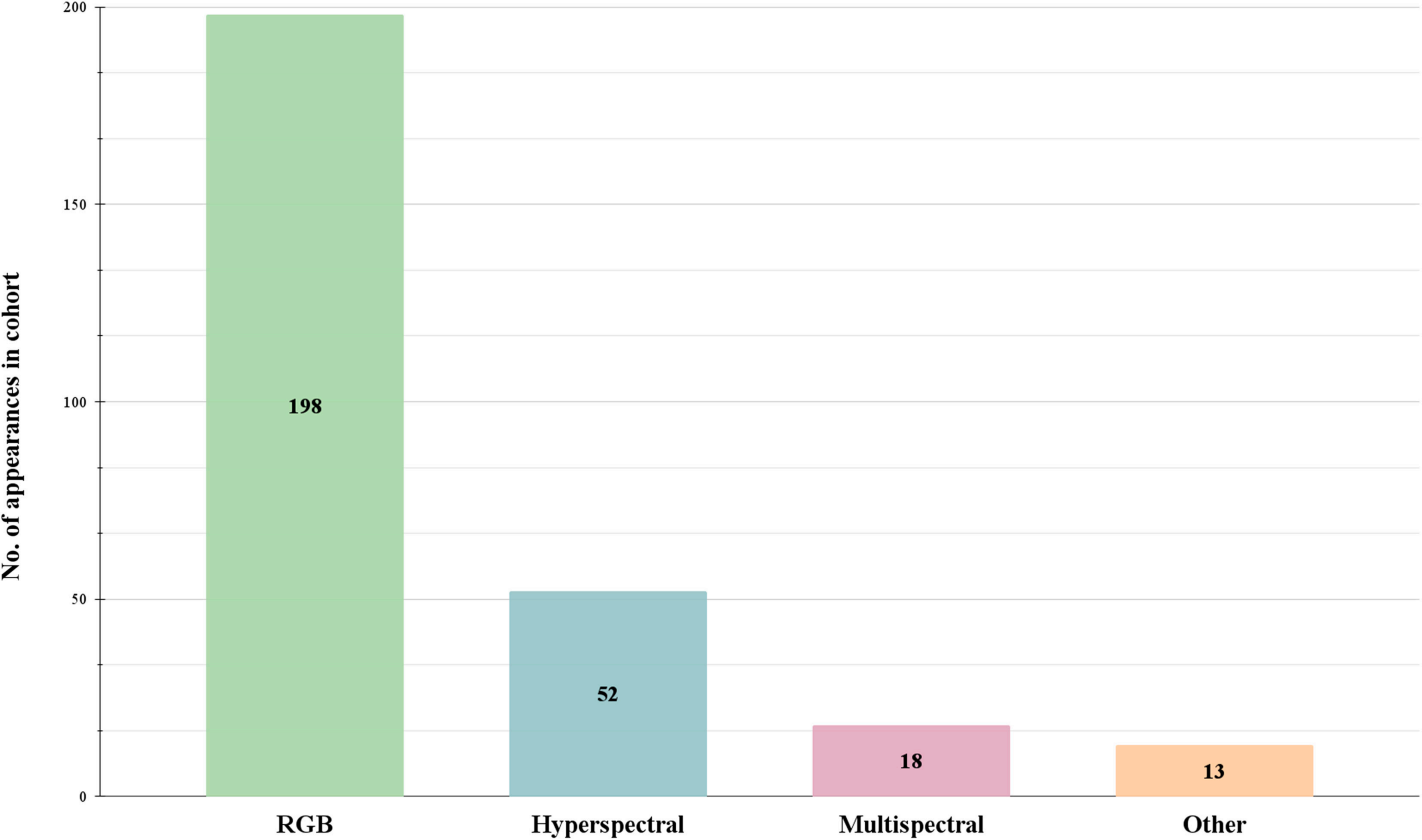
Overview of the total number of occurrences for each imaging sensor type: RGB in green; hyperspectral in blue; multispectral in purple, and other imaging sensors in orange. The other category includes imaging sensors with few occurrences, namely fluorescence, thermal, satellite, and LiDAR.

In this SLR, we recorded 198 instances of the RGB imaging sensor, making it the most popular sensor among the 262 studies (Fig. 4). We expected this result as RGB imaging sensors are more accessible and less expensive than spectral sensors [77]. On average, a low-cost RGB imaging sensor can be purchased for less than €100 providing researchers with the ability to image their plants. Raspberry Pi camera modules are a popular choice for researchers to create their imaging sensor by inserting it into a Raspberry Pi computer [78]. An example of using a Raspberry Pi was demonstrated in field-based conditions to image *Cercospora* leaf spot in okra together with a deep CNN algorithm [79]. Another popular RGB option is smartphone cameras that have become increasingly used in plant imaging due to their high pixel resolution and without the need for previous expertise.

**Fig. 5.**
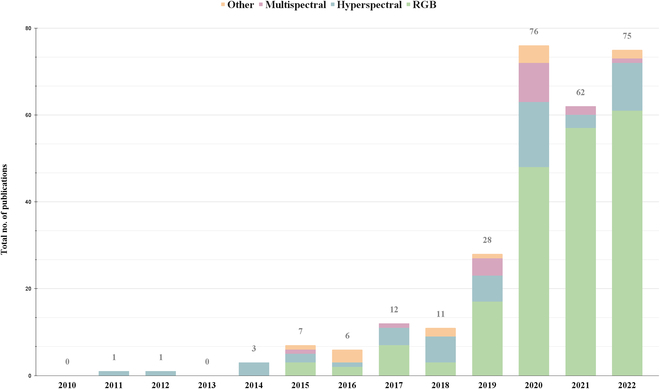
Summary of the total number of studies by their year of publication, along with the specific imaging sensor used for analysis in each study: RGB in green; hyperspectral in blue; multispectral in purple, and other imaging sensors in orange. The number of studies also includes published papers that employed multiple imaging sensors.

While many studies used RGB sensors, we also found that 52 studies adopted hyperspectral sensors, and 18 studies used a multispectral sensor. We observed that spectral sensors began to see a gradual increase from 2019 onward. A likely reason for these results is that spectral sensors are very expensive, and many researchers may lack the financial resources necessary, leading them to opt for the less expensive alternative (RGB imaging). These sensors also require technical proficiency and specialized software, as well as analyzing tools only available through premium subscriptions, limiting their use. Open-source libraries also exist but are still in the early stages of development. For example, SPy [80] and hsda [81] need further development to process spectral images and include user-friendly features. Despite these challenges, spectral imaging is a powerful tool when used to uncover stress-related symptoms that may not be easily observable with other imaging sensors. We hypothesize that the high number of studies using hyperspectral sensors since 2020 is mainly due to the complex nature of hyperspectral data, which requires AI to have a grasp on its biological meaning as well as its lower cost compared to previous years (Fig. 5).

From the 262 studies reviewed in our SLR, we observed a trend of limited research combining AI with various imaging technologies such as fluorescence, thermal, satellite imaging, and LiDAR. Despite their potential, these technologies have seen less integration with AI tools compared to RGB and spectral sensors.

• Fluorescence imaging: Among the studies, only 2 utilized fluorescence sensors in conjunction with AI. For instance, one study leveraged a pulse amplitude modulated chlorophyll fluorescence imaging system, FluoCam FC800, to image *Arabidopsis thaliana* plants under drought stress [82]. The study deployed a variety of AI algorithms such as linear discriminant analysis and k-nearest neighbor (KNN) to dynamically monitor the photosynthetic fingerprints caused by sos genes under drought conditions [82]. In conclusion, the authors found that both linear discriminant analysis and KNN achieved a high accuracy of 95.0% in differentiating between the genotypes based on the chlorophyll fluorescence data [82]. Thus, indicating the potential of using kinetic chlorophyll fluorescence imaging and AI algorithms to effectively monitor and classify plant responses to drought stress.

• Thermal imaging: Four studies incorporated thermal sensors with AI. A notable example involved one study using both RGB and thermal sensors to monitor powdery mildew stressed tomato plants [83]. Using an SVM classifier the study concluded that by combining information from thermal and RGB images, tomato plants infected with *O. neolycopersici* can be identified with high accuracy, more than 90% [83].

• Satellite imaging: Satellite imaging was used in conjunction with AI in 5 studies. One prominent study chose to test the capability of high spatial resolution multispectral satellite imagery, specifically the SPOT-6 sensor, to detect and map powdery mildew disease in winter wheat at a regional scale [84]. To achieve this goal the authors used a SAM to help detect and map the powdery mildew disease from the satellite imaging data. With a mapping accuracy of 78% and a kappa coefficient of 0.55, this suggests that the high-resolution multispectral satellite image data has practical potential in crop disease monitoring [84].

• LiDAR: Only one study in our review used LiDAR technology combined with AI. This research article focused on the use of terrestrial laser scanning (TLS) coupled with ML algorithms to detect and classify the severity of basal stem rot disease in oil palm plantations [85]. By using TLS the authors captured 3D point cloud data and preprocessed and each point cloud to extract features such as crown slice at different heights, crown area, frond angle, and frond number [85]. Ultimately, the authors combined these features with ML algorithms such as kernal naive Bayes which produced an accuracy of 85% and a kappa coefficient of 0.80 [85]. Thus, demonstrating how effective both TLS and ML can be in predicting early basal stem rot infection.

Our findings highlight the underuse of these advanced imaging technologies in AI-integrated plant stress research. While cost and specific technical challenges, such as the need for dark adaptation in fluorescence imaging, might contribute to their limited application, the examples from our review demonstrate their effectiveness in identifying stress symptoms. These instances suggest a growing, albeit slow, recognition of the value these technologies bring to the field, potentially leading to more widespread adoption and innovation in future research.

### Abiotic stress research remains uncharted territory with untapped potential

An unsurprising outcome of this SLR is the lack of studies focusing on abiotic stress. While we found several studies that opted to focus on abiotic stress (5%), the vast majority focused instead on biotic stressors. A likely reason for this result is the popularity of using RGB imaging sensors to capture traits associated with disease symptoms (e.g., discoloration or lesions). This makes RGB imaging a quick and precise tool for imaging biotic stressed plants, as reflected in 198 studies. On the other hand, abiotic stress presents a more subtle impact with RGB imaging, demanding a more complex examination of the data to interpret these responses (e.g., stunted growth, reduced leaf area). To overcome the challenge of data interpretation in abiotic stress, our results suggest that researchers selected advanced tools such as thermal, hyperspectral, or fluorescence imaging sensors. For example, drought stress can cause wilting and reduced growth, requiring longitudinal analysis of growth and architecture using RGB imaging. Yet, changes in stomatal conductance and leaf temperature are easily detected with thermal imaging sensors [86].

Despite the challenges and limitations of RGB imaging in abiotic stress, several studies have efficiently used these sensors. For example, a published paper targeted soybean, maize, and okra under field-based conditions, demonstrating how state-of-the-art DNNs, such as Inception V3, can predict drought symptoms [87]. In this SLR, 5 studies used RGB imaging to record color variations in leaves, enabling the detection and quantification of nutrient imbalances [87,88]. Most abiotic stress studies used spectral sensors for various stressors (e.g., salinity, heat, and drought). For example, hyperspectral imaging and SVM algorithms were used to detect early heat and water stress in strawberry plants [89]. Taken together, abiotic stress research using imaging sensors, particularly spectral sensors, and AI is still in its infancy. However, our results emphasize an untapped potential of this area to understand and predict the effects of the environment on plants [82,89].

### AI is revolutionizing how we analyze plant stress

**Fig. 6.**
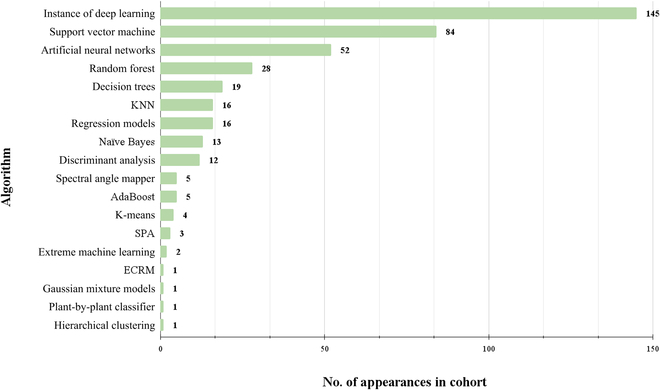
The total number of AI algorithms used in plant imaging studies in descending order. Each algorithm was recorded once for each study, even if the algorithm was used in multiple ways.

Here, we dissect the use of different AI algorithms and their trends in uptake throughout time (Fig. 6). To facilitate data collection regarding studies and the respective use of AI algorithms, we only recorded one DL algorithm per publication. A key result from this SLR was the breakdown of which studies used either ML or DL. We found that 115 studies used ML, while 135 opted exclusively for DL, indicating that DL has become a popular option for identifying plant stress symptoms, increasing from 9 studies in 2019 to 45 in 2022. Among the 262 primary studies, 146 utilized DL combined with another algorithm, corresponding to a small percentage of studies using ML and DL to create new and more complex models, demonstrating how researchers are bridging the gap between the 2 areas and doing so in interesting ways. For example, some studies used ML for feature extraction (e.g., plant height) and DL for classification tasks (e.g., susceptible or tolerant cultivar). The vice versa also exists where ML algorithms such as SVM are used to create predictions on features extracted by a DNN.

From our results, we can conclude that DL algorithms are nowadays the most common, with CNNs taking the top place in DL architecture. This is unsurprising, as CNNs are typically used for image classification and segmentation tasks as they excel in capturing features from visual data. For example, researchers used AlexNet and VGG-16 to identify maize and olive foliar diseases [90,91]. AlexNet and VGG-16 represent traditional CNN architectures; nevertheless, we can observe an increase in the use of more recent architectures such as MobileNet, YOLO, and DenseNet to identify mango and apple leaf disease and wheat rust [92–94]. Unsurprisingly, RGB sensors constitute 140 of the 145 studies that used DL (96%), which is due to RGB images capturing intricate patterns and subtle details at a low cost. For example, researchers have used smartphones combined with numerous algorithms, including InceptionV2, MobileNetv2-YOLOv3, and a self-structured CNN, to detect plant diseases [95–97]. Interestingly, we found that spectral imaging datasets are not commonly used for DL models. There are 2 main reasons for this. First, DL algorithms typically require large amounts of labeled data to learn and generalize effectively, which can be challenging and time-consuming, with open-source labeled datasets being uncommon. Second, DL models, such as CNNs, can be computationally intensive and require substantial computational resources for training. Nonetheless, given the rapid acceleration of DL research, we may predict that the use of DL for spectral data will see a major increase soon.

Our results show that ML algorithms are widely adopted in the area. We observed that SVM (84 studies), ANN (52), and random forest (28) are the most common AI algorithms. The SVM has been successfully used in various research tasks. SVMs are supervised ML algorithms used for classification and regression analysis. An SVM represents data in an *n*-dimensional space, where *n* corresponds to the number of features in the dataset (phenotypic traits), thus being very valuable in imaging studies where in one single day, a researcher can obtain dozens of traits. In the end, despite its simplicity, the SVM algorithm is capable of predicting the relevance of a trait compared to others in terms of stress response. For example, a modified version of SVM was used to study foliar diseases in rice, enabling the detection of 5 different diseases with 98.63% assurance [98]. SVM algorithms have also been used in conjunction with decision trees, logistics regression, naïve Bayes, ANN, and long short-term memory algorithms to detect the early onset of downy mildew in *Vitis vinifera* using thermal imaging technology [99]. These algorithms enabled authors to distinguish a single grapevine leaf infected or noninfected with an accuracy of 81.6%. They pinpointed that the best time of the day to take the image was between 10:40 AM and 11:30 AM [99].

ANNs have been extensively utilized (52 studies). ANNs are mathematical models that imitate the structure, function, and learning capacity of biological neural networks in the human brain. They are composed of artificial neurons that process information and make decisions collectively. Each artificial neuron receives input from another neuron, processes this data using a mathematical function known as an activation function, and transmits the resulting signal to other neurons. Supervised learning can be used to train ANN, where the network learns from a collection of input and output pairs. For example, Martínez-Martínez et al. [100] used an ANN coupled with principal component analysis to estimate leaf spot severity in common bean [100]. The combination of principal component analysis and an ANN facilitated RGB segmentation which allowed further detection and classification of images. Modified ANNs such as backpropagation neural networks and multilayer perceptrons have been utilized in multiple studies. For example, a feed-forward neural network with unsupervised learning was used to classify and predict when to apply treatment to cotton root rot symptoms [101].

Other AI algorithms such as random forest, KNNs, and decision trees have also been extensively used. Regression-based methods include regression and discriminant analysis models, such as logistic regression, elastic net regression, and various implementations of partial-least-squares discriminant analysis. For example, regression-based methods have been used to classify the severity of 5 plant disease lesions in symptomatic leaves by employing RGB images from the PlantVillage dataset in several species such as bell peppers, soybean, wheat, and potato, among others [102,103]. The application of regression models in AI is on the rise, with their use increasing from 3 studies between 2006 and 2021 to 10 studies in 2022, suggesting that regression-based methods offer a comprehensive evaluation of plant stress.

Our SLR concluded that unsupervised learning has been sporadically used as we only found 5 unsupervised learning studies to predict stress responses. The unsuitability of these models can explain this result to predict plant stress symptoms because supervised learning models can directly learn the discriminatory information from images and predict specific stress-related patterns. Nevertheless, unsupervised approaches can identify plant stress symptoms and can be a useful tool if the target image dataset is not labeled. Unsupervised methods include clustering algorithms such as k-means that separate a dataset into several groups. An example includes the work of Savian et al. [104], where thermal and multispectral imaging was used to detect Kiwifruit vine decline syndrome. By clustering the temperature data using k-means and hierarchical clustering, the researchers could predict a disease outbreak one year in advance, with an accuracy of 71% [104].

Altogether, we may conclude that AI is revolutionizing plant stress analysis and prediction, and we should understand the reasons behind the discernible breakdown of AI algorithms per imaging sensor. Our SLR established that studies using a DL architecture heavily relied on RGB sensors (140 studies), while only 6 used spectral imaging and DL. In contrast, 76 studies used ML in spectral sensors (66% of all ML studies). We also show that algorithms such as the SVM, SAM, and decision trees are becoming quite popular in processing spectral data. This result, however, does raise the question as to why ML algorithms are more frequently used for spectral datasets. We theorize that spectral imaging requires complex models to identify features (e.g., chlorophyll content), and ML models simplify the interpretation of the results, providing clear rules for decision-making. In contrast, DL models, known for their black-box nature, hinder the biological meaning behind their predictions. The limited availability of spectral imaging datasets, specifically open-source, is another reason behind the low use of DL models, which require large labeled datasets to learn and make accurate predictions. At present, the limited spectral data available has benefited from using ML algorithms, such as SVMs or random forests, to extract key stress-related traits and deliver accurate predictions swiftly. In the future, we speculate that the landscape will shift as spectral data becomes more abundant and accessible, allowing DL to demonstrate its full potential in spectral imaging and the accuracy of predictions.

## Conclusions and Future Directions

We used a 3-phase review strategy and programmable bots to thoroughly examine the use of AI and imaging sensors for plant stress analysis (abiotic and biotic) from 2006 to 2022 inclusive. With this breakthrough analysis, we found 2,704 published manuscripts in 4 databases (Springer, ScienceDirect, PubMed, and Web of Science), which specifically used imaging sensors and AI to interpret the data. The value output of this SLR is more than double the average output of current SLR strategies. Allowing us to retrieve 262 relevant studies, carefully curated and analyzed.

Our SLR began by posing 3 research questions which we can now answer clearly. Firstly, we asked about the “current status of the research on imaging sensors and AI for plant stress analysis?” We observed a shortage of publications before 2015, with the first surge in 2019 (26 studies), increasing to 65 in 2020 and 72 in 2022. By exploring this research question, we discovered a bias in research toward biotic stress (95% of studies). This fact can be attributed to the “easiness” of detection of visual symptoms due to the necrotic and chlorotic nature of disease lesions. Our results also acknowledge the strong contribution of open-source imaging datasets in advancing DL research and more accurate predictions. Thus, leads us to predict that DL will increase greatly due to an extended availability of open-source spectral datasets.

Secondly, we asked, “Which imaging sensors and AI are currently the most popular for plant stress analysis?” We found that RGB sensors are the most popular method (192 studies). However, hyperspectral imaging (52 studies) has quickly progressed since 2020. Our analysis also revealed that DL is currently the most popular algorithm (145 instances), with networks such as VGG-16, MobileNet and Inception V2 seeing vast usage. Nevertheless, we can also acknowledge the strong use of ML algorithms such as SVM (84), ANN (82), and random forest (28). Taken together, we expect to see greater use of spectral sensors in abiotic stress, with hyperspectral imaging taking the lead in glasshouse conditions. In contrast, multispectral imaging will become more frequent in field trials. As for the future of AI trends, we believe that a combination of networks will become commonplace. We also speculate that transfer learning will be further explored to transfer the knowledge of stress symptoms learned from one to another AI algorithm, enabling navigation prediction between different plant species [105–107].

Thirdly, we asked, “What are the emerging trends in imaging sensors in plant stress research?” We found that while RGB and spectral sensors dominate the field, other imaging technologies such as fluorescence, thermal, satellite, and LiDAR imaging are increasingly being explored in spite of their current limited scope. Fluorescence imaging, with its unique ability to highlight photosynthetic changes in plants, could see increased application when integrated with AI algorithms. Future research may explore the use of AI to interpret fluorescence signals more accurately, enabling the detection of subtle stress indicators and could lead to more sensitive and specific stress detection systems, useful in early-stage disease identification. Thermal imaging, while currently limited by its high cost and complexity, holds potential in AI-assisted irrigation management and disease prediction. Advanced AI models could be developed to interpret thermal data more effectively, helping in the precise identification of water stress and pest infestations across large agricultural landscapes. Satellite imaging is poised to benefit substantially from advancements in AI, particularly in large-scale monitoring of abiotic stress factors like drought and heatwaves. The integration of satellite data with AI algorithms could lead to more accurate and timely predictions of environmental stress impacts on crop health, aiding in global food security efforts. LiDAR technology, with its capability to provide detailed structural information, may find enhanced usage in combination with AI for 3D phenotyping of plants. This could lead to better understanding of the physical manifestations of stress and improve the accuracy of stress detection and phenotyping in complex environments.

Finally, we asked, “What limitations exist in current imaging sensors and AI algorithms for plant stress analysis?” RGB sensors are the main method used to develop new DL models due to their low cost and relatively simplistic technical knowledge requirements. Nevertheless, we demonstrate the ability of spectral sensors to act as promising tools for plant stress analysis, despite the current scarcity of datasets, which can cause limitations in prediction ability. Our results shed light on the low uptake of DL for spectral analysis (6 studies), with ML proving to be more popular in predicting stress responses (76 studies). Another explanation behind the limitation in the uptake of DL can be attributed to a need for steep computational resources apart from the limited availability of labeled datasets. We expect that future research fields will use explainable AI to study plant stress, as researchers will be able to understand the reasoning behind the selection of an algorithm, i.e., the biological meaning of a decision [108,109]. Moreover, with current advances in LiDAR and 3D imaging, we foresee greater use of DL with more complex architectures to analyze stress symptoms [85]. In conclusion, our SLR provides an overview of the current state-of-the-art uses of AI and imaging sensors for plant stress analysis.

## Data Availability

All code used to create and run the programmable bots is available on GitHub (https://github.com/Walshj73/data-processing-bot.git) and licensed under the MIT license. All 262 studies found during this SLR process are available in a publicly accessible Zotero group library (titled “Advancements in Imaging Sensors and AI for Plant Stress Detection”). The group library can be accessed on Zotero by using the “Search for groups” feature found under the group’s tab and searching for “Advancements in Imaging Sensors and AI for Plant Stress Detection: A Systematic Literature Review”. Membership to this group is free and publicly accessible to all.
