# Supplementary figures and images for: Advancements in Imaging Sensors and AI for Plant Stress Detection: A Systematic Literature Review
Source: Plant Phenomics. 2024 Mar 1;6:0153. doi: 10.34133/plantphenomics.0153 (PMC10905704; doi:10.34133/plantphenomics.0153)

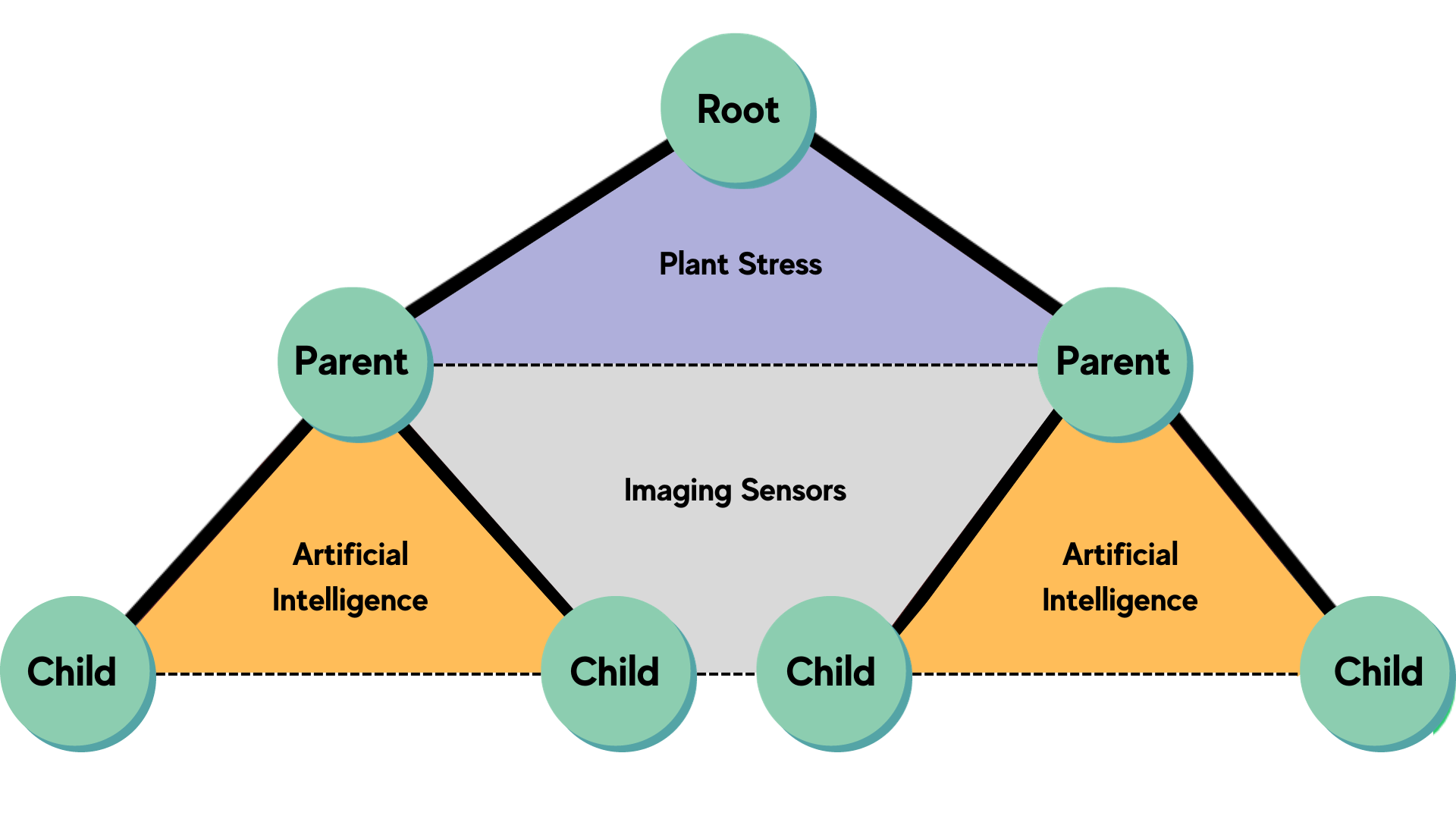

Supplement: Supplementary 1 — Figs. S1 to S4 [file plantphenomics.0153.f1.zip › Supplemental Figure 01.png]

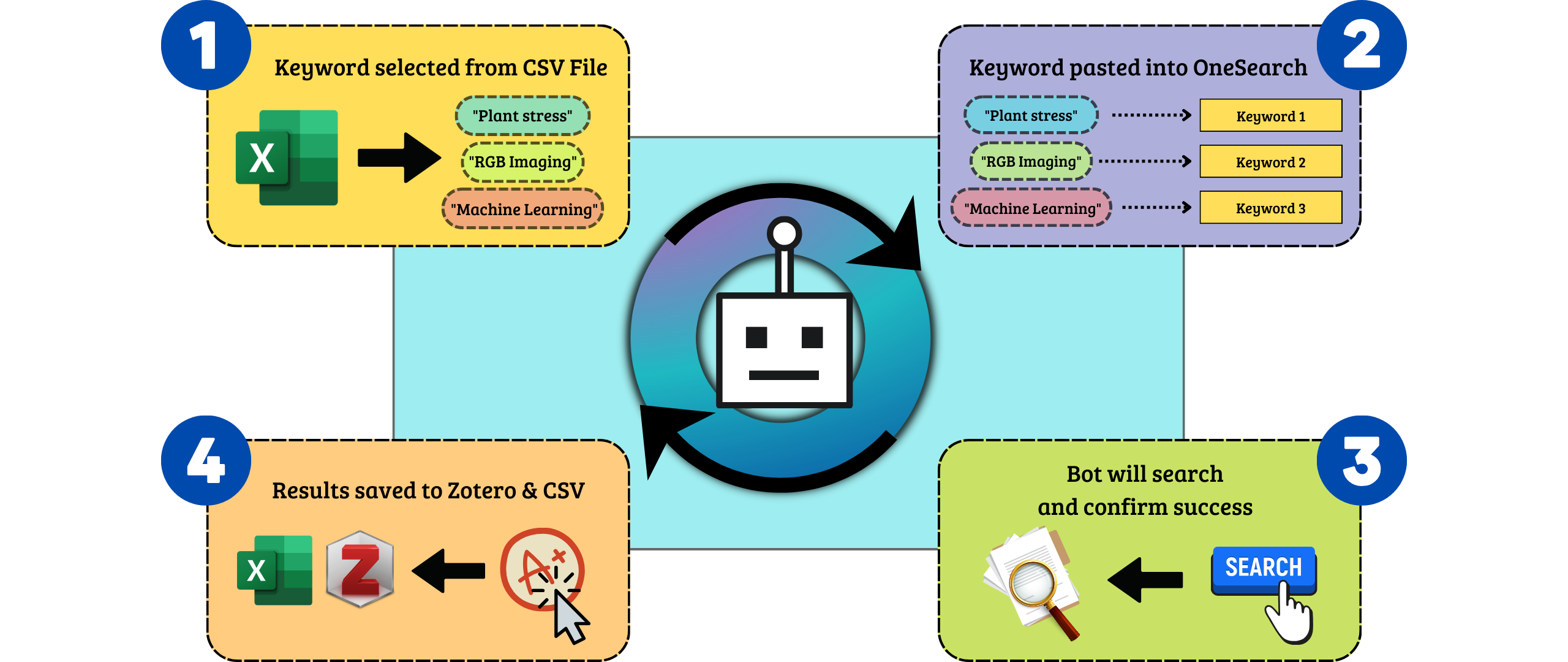

Supplement: Supplementary 1 — Figs. S1 to S4 [file plantphenomics.0153.f1.zip › Supplemental Figure 02.png]

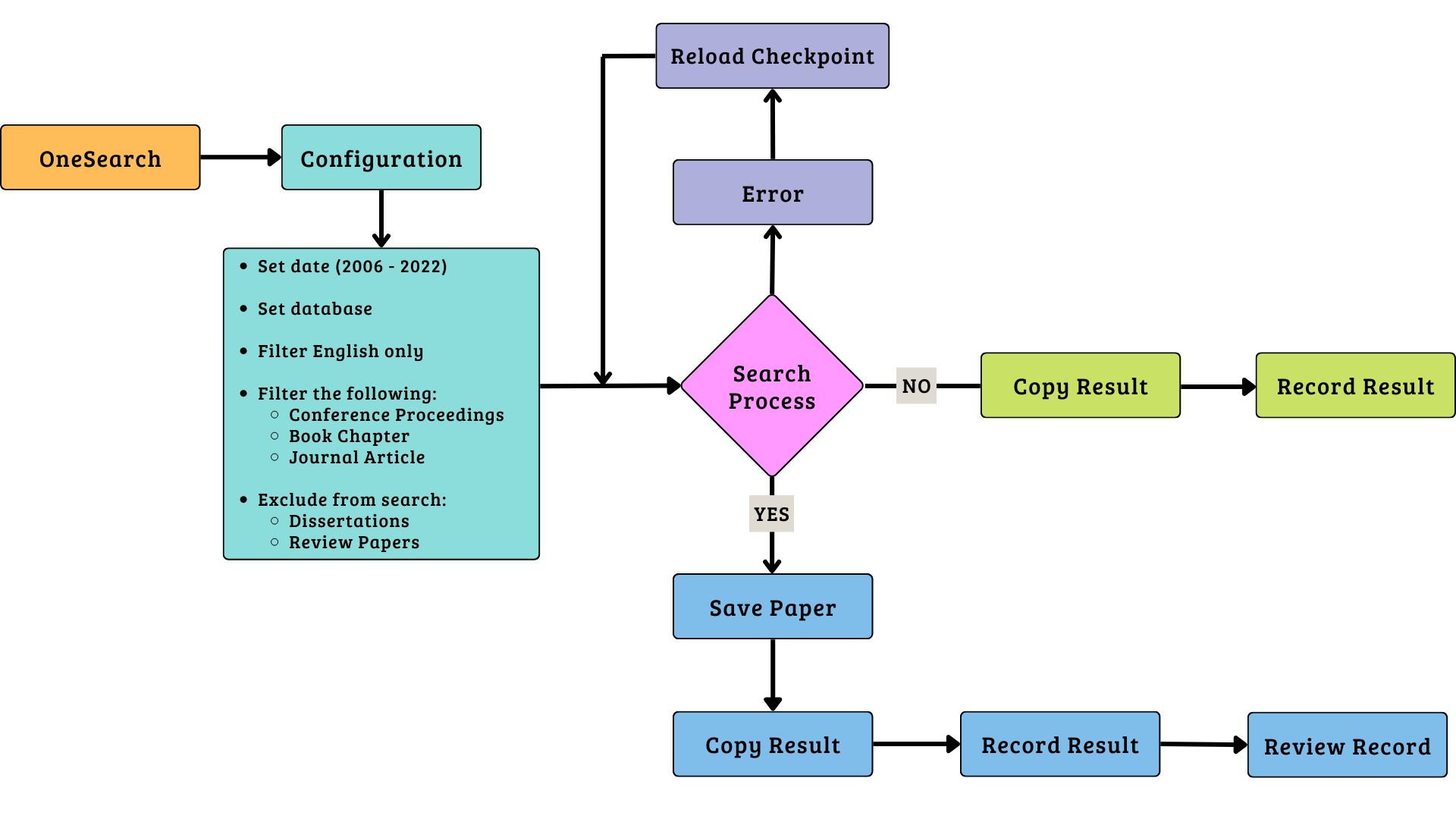

Supplement: Supplementary 1 — Figs. S1 to S4 [file plantphenomics.0153.f1.zip › Supplemental Figure 03.png]

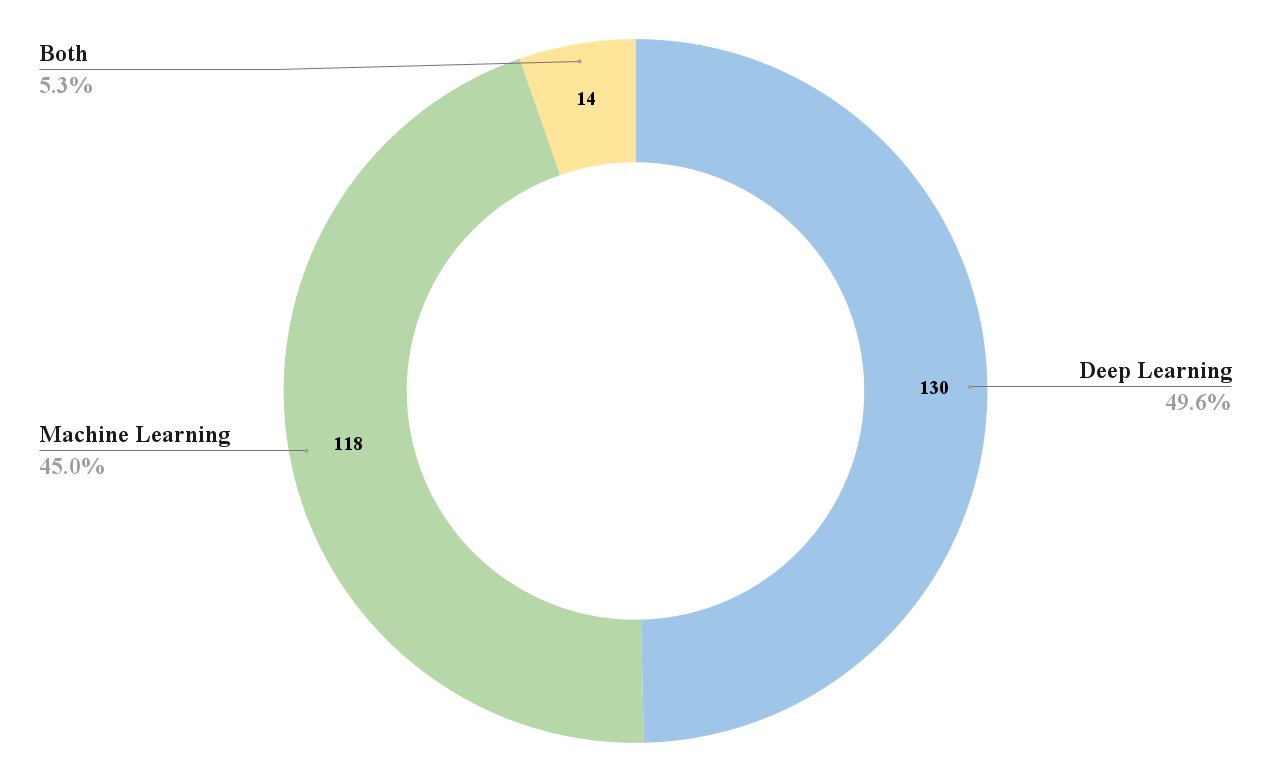

Supplement: Supplementary 1 — Figs. S1 to S4 [file plantphenomics.0153.f1.zip › Supplemental Figure 04.png]
